# DNA Variation in the *SNAP25* Gene Confers Risk to ADHD and Is Associated with Reduced Expression in Prefrontal Cortex

**DOI:** 10.1371/journal.pone.0060274

**Published:** 2013-04-12

**Authors:** Ziarih Hawi, Natasha Matthews, Joseph Wagner, Robyn H. Wallace, Tim J. Butler, Alasdair Vance, Lindsey Kent, Michael Gill, Mark A. Bellgrove

**Affiliations:** 1 Queensland Brain Institute, the University of Queensland, Brisbane, Australia; 2 The Royal Children's Hospital, University of Melbourne, Victoria, Australia; 3 School of Medicine, University of St Andrews, St Andrews, Scotland, United Kingdom; 4 Departments of Psychiatry, Trinity College Dublin, Dublin, Ireland; 5 School of Psychology and Psychiatry, Monash University, Melbourne, Australia; University of Wuerzburg, Germany

## Abstract

**Background:**

The Coloboma mouse carries a ∼2 cM deletion encompassing the *SNAP25* gene and has a hyperactive phenotype similar to that of ADHD. Such mice are 3 fold more active compared to their control littermates. Genetic association studies support a role for allelic variants of the human *SNAP25* gene in predisposing to ADHD.

**Methods/Principal Findings:**

We performed association analysis across the *SNAP25* gene in 1,107 individuals (339 ADHD trios). To assess the functional relevance of the *SNAP25*-ADHD associated allele, we performed quantitative PCR on post-mortem tissue derived from the inferior frontal gyrus of 89 unaffected adults. Significant associations with the A allele of SNP rs362990 (χ^2^ = 10, p-corrected = 0.019, OR = 1.5) and three marker haplotypes (rs6108461, rs362990 and rs362998) were observed. Furthermore, a significant additive decrease in the expression of the SNAP25 transcript as a function of the risk allele was also observed. This effect was detected at the haplotype level, where increasing copies of the ADHD-associated haplotype reduced the expression of the transcript.

**Conclusions:**

Our data show that DNA variation at *SNAP25* confers risk to ADHD and reduces the expression of the transcript in a region of the brain that is critical for the regulation of attention and inhibition.

## Introduction

Attention deficit hyperactivity disorder (ADHD) is a highly heritable disorder of childhood with significant functional impairment and negative lifetime outcomes across all developmental stages. The disorder is marked by disruption of catecholamine signaling, with mainstay treatments for the disorder targeting the dopamine and noradrenaline transporters and the alpha 2a adrenoreceptor [1]. Although a focus on these molecular targets has proven informative for genetic association, identifying the molecular machinery driving neurotransmitter release may provide critical insights into the biology of ADHD.

Synaptosomal-associated protein (SNAP-25) is a presynaptic plasma membrane protein that is specifically and highly expressed in nerve cells. The gene encodes a protein component, which interacts with the membrane associated protein syntaxin, and vesicle-associated membrane protein (VAMP) to form the SNARE complex. This complex interacts with membrane proteins known as synaptotagmin to make up a core complex essential for docking and holding synaptic vesicles at the presynaptic membrane in preparation for Ca^2+^-triggered neurotransmitter exocytosis [2].

The Coloboma mouse bears a semi-dominant mutation (cm/+) in which the heterozygous form results in the mutant type while the homozygous is lethal. The mutation is a ∼2 Cm deletion encompassing genes including *SNAP25* and the gene encoding phospholipase C beta (*PLCB-1*) [3], [4], mapping to human chromosome 20 [5]. The Coloboma mouse is considered an animal model of ADHD as it displays a hyperactive phenotype that is reduced by stimulant drugs such as dextroamphetmaine [6]. An increase of up to 40% in noradrenaline within the striatum and the nucleus accumbens of the Coloboma mouse has been reported [7]. Furthermore, mRNA expression of Tyrosine Hydroxylase (a rate limiting enzyme in the synthesis of dopamine and noradrenaline) was significantly increased in the cells of the noradrenergic locus coeruleus, indicating that a perturbed level of noradrenaline (presumably caused by the lack of *SNAP25* copy) may contribute to the hyperactive behavior of the *coloboma*. Similarly, NA depletion using DSP-4(N-(2-chloroethyl)-N-ethyl-2 bromobenzylamine hydrochloride) significantly reduced hyperactivity in the Coloboma mouse, supporting the noradrenaline hypothesis [8].

Genetic association studies suggest that allelic variations in the *SNAP25* gene might confer susceptibility to ADHD. An initial analysis [9] on two single nucleotide polymorphisms (SNPs) (rs3746544 and rs1051312) located at the 3′untranslated (3′UTR) region of the gene found a trend toward excess transmission of the C allele of rs1051312. Significant association of the above 2-SNP haplotype was reported [10], [11]. Brophy et al. [11] reported preferential transmission of allele T of rs1051312 to Irish ADHD cases. Two other studies have shown association with ADHD using a microsatellite marker in intron 1 and marker rs363006 mapped to intron 7 of the gene [12], [13]. Other SNPs within the gene have also been reported to associate with ADHD in more recent studies. Feng et al. [14] observed association with SNPs rs6039806, rs362549, rs362987 and rs362988, mapped to introns 3, 4, 5 and 7 respectively, while Kim et al. [15] reported association with SNP rs3787283 (intron 7), which is in strong linkage disequilibrium (LD) with the 3′UTR SNPs rs3746544 and rs1051312 (D′ = 0.89–0.94). Association with rs3746544 has also been reported in Korean [16] and Eastern Indian ADHD samples (17]. Other studies however failed to observe significant association between SNAP-25 variants and ADHD [18], [19]. A recent meta-analysis demonstrates that there is considerable evidence to support an association between ADHD and DNA variation in the *SNAP25* gene [20]. Together, these findings provide promising evidence that SNAP-25 is likely to contribute to the development of ADHD and that the region between intron 3 and the 3′UTR may contain a variant(s) affecting gene's expression.

Here we sought to provide additional evidence for a role of SNAP-25 in ADHD by performing dense SNP mapping across the gene in nuclear families with ADHD. To assess the functional relevance of ADHD-associated variants, we performed quantitative PCR (qPCR) of the SNAP-25 transcript in a large sample (N = 89) of post-mortem brains from non-clinical individuals. Our qPCR work focused on expression of the transcript in human inferior frontal gyrus (IFG), an area of the cortex that has consistently been implicated in ADHD from both structural and functional imaging analyses [21], [22]. We show that ADHD-associated variants of *SNAP25* influence expression within the IFG, with decreased SNAP-25 expression (as expected from the Coloboma mouse) associated with the ADHD risk variants.

## Materials and Methods

### ADHD participants

A total sample of 1,017 individuals, comprising 339 Caucasian ADHD probands and their parents (full trios) across three similar sub-samples was investigated. The first cohort comprised 185 full trios recruited throughout Ireland from child psychiatric clinics and schools in West County Dublin and from the Hyperactive and Attention Deficit Children's Support Group of Ireland. Consensus diagnoses were made according to DSM-IV ADHD either with or without comorbidity. These diagnoses were based on all available clinical information and the Child Behaviour Checklist (CBCL), the Conners' Parents and Teachers Rating Scales, and the Comprehensive Teachers Rating Scale (ACTeRS) [23]. The second sample comprised 68 Australian nuclear families with ADHD recruited from the Royal Children's Hospital Melbourne and the Queensland Brain Institute, Brisbane. This sample shared diagnostic, inclusion and exclusion criteria with the Irish sample [24]. The third sample was ascertained from several child psychiatric clinics in the United Kingdom. It comprised 86 trios where the child met DSM-IV diagnostic criteria for ADHD, as assessed using the same instruments as for the Irish cases [25]. Across all three cohorts, the mean age of the participants was 10.53 years (*SD* = 3.67) and were predominantly male (87%). All probands fulfilled DSM-IV diagnostic criteria for ADHD. Of these, *N* = 44 (13%) had the inattentive subtype, and *N* = 19 (5.6%) had the hyperactive impulsive subtype; the remainder had the combined subtype (81.4%). One hundred and seventy-nine probands (52.8%) had a comorbid diagnosis of oppositional defiant disorder (ODD) and 34 (10%) fulfilled criteria for comorbid conduct disorder (CD). Frequencies of ADHD subtypes and comorbidities were similar across the recruitment sites. We obtained a written consent from the next of kin, caretakers, or guardians on the behalf of the minors/children participants involved in our study. The consent was in accordance with the declaration of Helsinki and the ethical approvals from the University of Queensland, Australia, Trinity College Dublin, Ireland, and the University of Birmingham, UK”

### Non-pathological brain samples

In addition to the ADHD samples, 89 unaffected Caucasian brain samples (inferior frontal gyrus; IFG) were obtained from the Australian Brain Bank. Of these, 63 (71%) were from male subjects. None of these individuals were diagnosed with ADHD or other psychiatric condition. The mean age of the sample was 51.6 years (SD = 12.2), PH ranged from 5.8–7 and the post-mortem interval (PMI) was 28.2 hours (SD = 14).

### SNP selection and genotyping

Fifteen SNPs were included in the current investigation (Table 1). SNPs were selected using Haploview default SNP tagging criteria. In addition, some SNPs that had been implicated in ADHD from previous studies (rs363020, rs3746544 and rs8636) were also included. Genotyping was commercially performed at the Australian Genome Research Facility (AGRF) using iPLEX GOLD chemistry with a Sequenom MassArray on an Autoflex Spectrometer (Sequenom, San Diego, CA). The genotyping success rate for all SNPs ranged between 96.7–98.9%. Genotyping of SNAP-25 SNPs rs362562 and rs362990 of the non-clinical brain samples was conducted using TaqMan assays C_11682_10 and C_2488333_10 respectively. SNP rs6108461 was genotyped using a standard PCR-RFLP assay. This was conducted using the forward primer 5′ CTTGAAGCATCCCAGGAAGA and the reverse 5′ GAAGGAAAAATGTTGGGGTTT 3′. The PCR product (214 bp) was then restricted with the enzyme Cac81 and the DNA fragments were fractionated on a 3% agarose gel. The G allele was characterized by 164 bp and 51 bp fragments while the A allele was characterized by 215 bp fragment.

**Table 1:** TDT analysis of *SNAP25* polymorphisms in ADHD nuclear families.

SNP	Allele	T	NT	?^2^	p-value	Ep-value*	OR	SNP position
rs3787303	T	74	73	0.007	0.9343	1.0	1.01	intron 1
rs363012	A	132	128	0.062	0.8041	1.0	1.03	intron 1
rs363039	G	153	151	0.013	0.9087	1.0	1.01	intron 1
rs12626080	G	148	145	0.031	0.8609	1.0	1.02	intron 1
rs363052	G	100	97	0.046	0.8307	1.0	1.03	intron 1
rs363020	T	75	66	0.574	0.4485	0.99	1.13	intron 1
rs362562	G	172	138	3.72	0.0535	0.41	1.25	intron 1
rs6108461	A	186	136	7.764	0.0053	0.06	1.37	intron 3
rs362990	A	150	100	10	0.0016	0.02	1.5	intron 4
rs362998	C	40	32	0.889	0.3458	0.99	1.25	exon 6
rs6039820	C	152	136	0.889	0.3458	1.0	1.11	intron 6
rs6108464	T	165	159	0.111	0.7389	1.0	1.04	intron 6
rs3787283	A	146	136	0.355	0.5515	1.0	1.07	intron 6
rs3746544	C	162	143	1.184	0.2766	0.97	1.13	3′UTR
rs8636	T	174	149	1.935	0.1642	0.86	1.18	3′UTR

T =  Transmitted, NT =  Not Transmitted, * =  Empirical P-value from HAPLOVIEW assessed the gene-wide significance value estimated on the basis of 10000 permutations.

### qPCR and SNAP-25 expression

Approximately 100–200 mg of inferior frontal gyrus (IFG) tissue of each sample was used to extract RNA and DNA using TRIZOL reagent as recommended by the manufacturers (Invitrogen). As PH is considered a good indicator of RNA purity and integrity, this was measured and found to range from 5.8–7. The optical density (OD) at 260/280 of RNA preparations ranged from 2–2.1 indicating good quality of the RNA preparation.

To minimize RNA contamination with the DNA, samples were treated with DNASE-I and cleaned using RNeasy Mini Kit as recommended by manufacturers (Qiagen, Doncaster, Victoria, Australia). Furthermore, to prevent any possible interference by DNA contamination when conducting qPCR, the target (*SNAP25*) and the reference genes β2micoglobulin (*β2M*) and beta actin (ACTB) gene primers were designed to amplify DNA regions involving exon 7 and 8 of *SNAP25,* exons 1 and 2 in *β2M and* exon 3 and 4 of *ACTB*. The qPCR primers were designed using the INTEGRATED DNA TECHNOLOGIES Oligo Design Tool, which is freely available at http://www.idtdna.com/scitools/scitools.aspx. The *SNAP-25*-qPCR forward primer sequence was 5′ATGGATGAAAACCTAGAGCAGG 3′ and the reverse was 5′ACACTTAAC CACTTCCCAGC 3′. The *β2M* forward primer sequence was 5′ GGCATTCCTG AAGCTGACAG 3′ whereas the reverse was 5′TGGATGAAACCCAGACACATAG 3′. The *ACTB* qPCR forward primer was 5′ACCACACCTTCTACAATGAGC 3′ and the reverse was 5′ GCGTACAGGGATAGCACAG3′. In this context, *β2M* and *ACTB* were used as reference genes as they have stable gene expression in various tissues including brain. A standard Invitrogen procedure was used to synthesize first cDNA strands of the samples. Relative quantification was performed using *SNAP25* (target gene) compared to *β2M* and *ACTB* (reference genes). PCR cycling was performed on a Roche LightCycler-480. Cycling conditions were 95°C for 5 min followed by 45 cycles at 95°C for 10 sec, 60°C for 10 sec and 72°C for 20 sec. This was followed by a melting curve cycle at 95°C for 5 sec and 1 min at 65°C. mRNA relative was expression quantified using normalized threshold cycles (Ct) of target relative to reference genes.

### Statistical analysis

Assessment of Hardy Weinberg Equilibrium for all SNPs was undertaken using parental DNA of the ADHD participants. All genotypes were in Hardy Weinberg equilibrium. Genetic association across a 15 SNP array was performed using the transmission disequilibrium test (TDT). Haplotype analysis to test for the transmission of multi locus haplotypes was performed by applying the default block definition in HAPLOVIEW (http://www.broad.mit.edu/mpg/haploview) using the method of Gabriel et al. [26]. A SNP block is formed if 95% of informative comparisons are in strong LD. Associations between ADHD risk variants and SNAP-25 expression in post-mortem IFG tissue was tested using linear regressions with an additive model (0 vs. 1 vs. 2 copies of the risk variant). In addition, possession (0 vs. 1 vs. 2 copies) of the risk or protective haplotypes was also assessed using linear regression.

## Results

### Genetic association

Fifteen SNPs were examined at the *SNAP25* gene covering a region of 88,6 kbp. TDT analyses of all individual SNPs are presented in Table 1. Two SNPs rs6108461 and rs362990, showed significant nominal association with ADHD (rs6108461: χ^2^ = 7.76, p = 0.0053, OR = 1.37; and rs362990: χ^2^ = 10, p = 0.0016, OR = 1.5). Permutation testing (10,000 permutations) revealed that only rs362990 (χ^2^ = 10, p-corrected = 0.019, OR = 1.5) was associated with ADHD, with a trend towards association for rs6108461 (p-corrected = 0.059).

**Figure 1:**
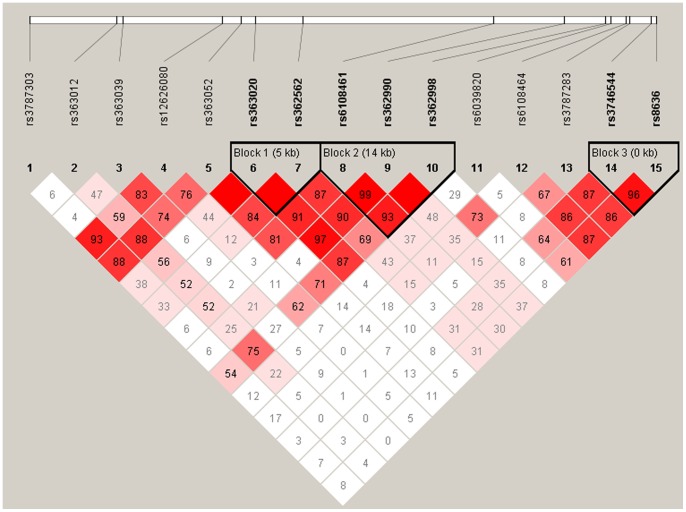
Linkage disequilibrium relation (expressed as D′ values) of *snap25* examined SNPs.

Although strong LD is apparent across the gene (Figure 1), three distinct blocks of LD were evident, with SNPs rs363020 and rs362562 form the first block and SNPs rs6108461, rs362990, rs362998 forming the second. The third block comprises the SNPs rs3746544 and rs8636. Haplotype analysis (Table 2) within these blocks showed significant association of a predisposing haplotype made of alleles AAC of the second block (χ^2^ = 8.37, p-corrected  = 0.019, OR = 1.4). An exploratory haplotype analysis using sliding window of 8 SNPs (∼20 kbp) extending from intron 3 (rs6108461) to the 3UTR (rs8636) enhanced the risk association signal (χ^2^ = 12.7, p = 0.0004, p-corrected  = 0.003, OR = 1.62). Finally, a haplotype comprised of the alleles GTC in the second haplotype block (see Figure 1) was less transmitted to ADHD cases than expected by chance (χ^2^ = 11.99, p-corrected  = 0.0019, OR = 0. 65). This implies that this haplotype may confer protection against the development of ADHD.

**Table 2:** Haplotype analysis of *SNAP25* SNPs in ADHD nuclear families.

Haplotype	Freq.	T	UT	?^2^	p value	Empirical p value*
Block 1						
AG	0.455	168	145	1.681	0.1947	0.865
AA	0.411	146	180	3.602	0.0577	0.317
TG	0.135	82	70	0.842	0.359	0.953
Block 2						
AAC	0.543	193	140	8.372	0.0038	0.0198
GTC	0.258	104	160	11.99	5.00E−04	0.0019
GAC	0.139	87	80	0.597	0.4395	0.986
GAT	0.056	33	40	0.78	0.3771	0.961
Block 3						
AC	0.624	151	169	1.002	0.3169	0.94
CT	0.358	172	149	1.682	0.1947	0.865

Freq =  Haplotype frequency, T =  Transmitted, UT =  Untransmitted, * = 10000 permutation.

### Functional analysis (qPCR)

**Figure 2:**
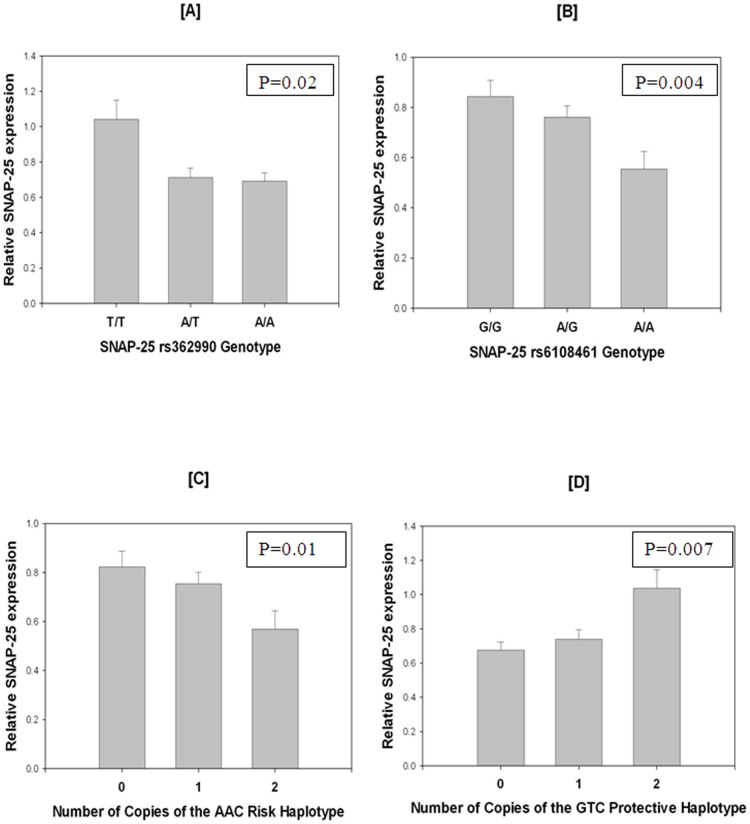
Relative expression of SNAP25 in non- pathological samples. A and B show decreased expression with ADHD associated alleles; C and D show the level of expression relative to ADHD risk and protective haplotypes respectively. Error bars represent the standard error of the mean.

A significant association between rs362990 and relative expression of the SNAP-25 transcript was observed (Figure 2). Specifically, increasing copies of the ADHD-associated A allele were associated with decreased SNAP-25 expression [F(1,84)  = 5.5, p = 0.02; R^2^ change  = 5.6.2%]. Similar results were found for rs6108461 [F(1,84)  = 8.9, p = 0.004; R^2^ change  = 8.7%]. The strong LD between these SNPs meant that controlling for one removed the effect of the other in the above analyses. Possession of the ADHD risk haplotype (AAC) was also significantly associated with expression of SNAP-25 in the IFG tissue [Figure 2C; F(1,84)  = 6.3, p = 0.01; R^2^ change  = 6.3%]. As expected, increasing possession (0 vs. 1 vs. 2 copies) of the risk haplotype was associated with decreased expression. Conversely, increasing possession of the protective haplotype was associated with increased SNAP-25 expression [Figure 2D; F(1,84)  = 7.8, p = 0.007 R^2^ change = 7.7%].

## Discussion

The results of the current study show that DNA variants of the *SNAP25* gene that associate with ADHD are also associated with functional changes in the expression level of the transcript in a region of the brain that is an established pathological locus for ADHD. The current investigation provides further support for the notion that *SNAP25* is a genetic risk factor for ADHD. Although nominal associations with two *SNAP25* SNPs (rs6108461and rs362990) were observed, only the association with rs362990 survived permutation testing. Haplotype analysis enhanced the association (OR = 1.62), suggesting that the region between intron 3 and the 3′UTR of *SNAP25* may harbor an important susceptibility variant for ADHD.

SNAP-25 is important for axonal growth and synaptic plasticity, which are essential steps for wiring the nervous system. Selective inhibition of SNAP-25 expression prevents neurite elongation of cortical neurons [27]. In addition, a high level of SNAP-25 expression in the adult brain was found to contribute to nerve terminal plasticity [28]. Furthermore, SNAP-25 functions in docking and fusion of synaptic vesicles in presynaptic neurons, which are essential for the regulation of neurotransmitter release. These functions make *SNAP25* an important candidate gene for ADHD.

Our results are consistent with some but not all previous association/linkage studies. Apart from the current investigation, a number of studies have tested for association between *SNAP25* gene variants and ADHD. All these studies have examined multiple SNPs and tested for association of single SNPs and haplotypes. The associated SNPs and their position within the *SNAP25* gene are presented in Table 3. Careful inspection of the data shows that ADHD-associated SNPs are clustered into two main regions. The first region, supported by eight studies, encompasses a region of 29 kbp extending from rs6039806 to rs8636 and maps between intron 3 and the 3′UTR of the gene. The second region, supported by five studies, extends from rs363032 to rs362569 spanning a region of 28 kbp and is mapped to intron 1. The associated SNPs in the current study (rs6108461and rs362990) map to introns 3 and 4. Interestingly, there are strong LD relations between these SNPs and those reported to associate with ADHD (except those mapped downstream of *SNAP25*; see Table 3). Furthermore, extended haplotype analysis comprising eight SNPs located between intron 3 (rs6108461) and the 3UTR (rs8636) enhanced the association (χ^2^ = 12.7, p-corrected = 0.003, OR = 1.62) indicating that this region may harbor a risk/functional variant for ADHD.

**Table 3:** Linkage disequilibrium relation of previously associated ADHD-*SNAP25* variants with SNPs of this investigation.

Previous Study	Associated SNPS	position	Current investigation	D′	r^2^
Barr et al. [9]; Kustanovich	rs3746544	3′UTR	rs6108461	0.40	0.11
et al. [10] and Brophy et al. [11]	rs1051312	3′UTR	rs6108461	ND	ND
Mill et al. [13]	rs363006	Intron 7	rs6108461	1.0	0.27
Feng et al. [14]	rs6039806	Intron 3	rs6108461	1.00	1.00
	rs362549	Intron 4	rs6108461	1.00	1.00
	rs362987	Intron 5	rs6108461	1.00	1.00
	rs362988	intron 7	rs6108461	1.00	0.10
Brookes et al. [35]	rs363020	Intron 1	rs6108461	1.00	0.18
Kim et al. [15]	rs3787283	Intron 7	rs6108461	0.19	0.01
Guan et al. [36]	rs8636	3′UTR	rs6108461	0.40	0.11
Elia et al. [19]	rs363032	Intron 1	rs6108461	0.26	0.04
	rs6133852	Downstream	rs6108461	1.00	0.05
Mick et al. [31]	rs362562	Intron 1	rs6108461	0.91	0.74
	rs362569	Intron 1	rs6108461	0.92	0.78
	rs362564	Intron 1	rs6108461	0.93	0.37
Sarkar et al. [17]	rs362569	Intron 1	rs6108461	0.92	0.78
	rs362988	Intron 7	rs6108461	0.54	0.20
Lasky-Su et al. [30]	rs363012	Intron 1	rs6108461	0.58	0.09
	rs363043	Intron 1	rs6108461	0.8	0.31
	rs362547	Intron 1	rs6108461	0.92	0.79
	rs1984830	Down stream	rs6108461	0.20	0.016
	rs6032846	Down stream	rs6108461	0.018	0.0
Barr et al. [9]; Kustanovich	rs3746544	3′UTR	rs362990	0.63	0.08
et al. [10] and Brophy et al. [11]	rs1051312	3′UTR	rs362990	ND	ND
Mill et al. [13]	rs363006	Intron 7	rs362990	1.00	0.06
Feng et al. [14]	rs6039806	Intron 3	rs362990	1.00	0.27
	rs362549	Intron 4	rs362990	1.00	0.27
	rs362987	Intron 5	rs362990	1.00	0.27
	rs362988	Intron 7	rs362990	0.51	0.11
Brookes et al. [35]	rs363020	Intron 1	rs362990	1.00	0.04
Kim et al. [15]	rs3787283	Intron 7	rs362990	0.39	0.09
Guan et al. [36]	rs8636	3′UTR	rs362990	0.63	0.08
Elia et al. [19]	rs363032	Intron 1	rs362990	0.01	0.00
	rs6133852	Downstream	rs362990	0.31	0.01
Mick et al. [31]	rs362562	Intron 1	rs362990	0.89	0.25
	rs362569	Intron 1	rs362990	1.00	0.30
	rs362564	Intron 1	rs362990	1.00	0.12
Sarkar et al. [17]	rs362569	Intron 1	rs362990	1.00	0.30
	rs362988	Intron 7	rs362990	0.51	0.11
Lasky-Su et al. [30]	rs363012	Intron 1	rs362990	0.49	0.017
	rs363043	Intron 1	rs362990	0.04	0.001
	rs362547	Intron 1	rs362990	1.00	0.30
	rs1984830	Downstream	rs362990	0.43	0.023
	rs6032846	Down stream	rs362990	0.27	0.14

ND =  LD Not defined.

Furthermore, it is important to emphasize that the quartile–quartile (QQ) plot for ADHD genome wide association (GWAS) for SNPs in or near candidate genes [29] showed mild inflation in the p-value distribution, providing evidence for association (although not significant at GWAS level). For *SNAP25*, several SNPs showed evidence of association with the symptoms of ADHD in a quantitative GWAS [30]. Three of these SNPS (rs363012, rs363043 and rs362547) are mapped to intron 1 of the gene. Interestingly, rs363043 and rs362547 (associated with total ADHD symptoms and with inattentive symptoms, respectively) (Lasky-su; personal communication) have strong LD with the ADHD-associated SNPs of the current study (Table 3) which further emphasises the importance of our findings. In addition, a recently published ADHD-GWAS reported evidence of association with 4 *SNAP25* markers including rs362562 [31]. The latter SNP is in strong LD with our SNPs (rs6108461: D′ = 0.91; rs362990: D′ = 0.89) and showed a trend toward association in our sample (Table 1) further confirming the importance of *SNAP25* variations in ADHD.

Computer simulation [32] suggests that the 3′UTR SNPs rs3746544 and rs1051312 (map within the associated region of this study) may alter the binding site of microRNAs (miR-510- miR-641) and consequently influence the level of SNAP-25 expression. Indeed, the post-mortem work reported herein hints at a potential pathophysiological mechanism by which *SNAP25* variants may increase risk to ADHD. Specifically, the lowered expression of SNAP-25 in regions of the cortex that are critical for attention and inhibition, such as the IFG, may ultimately decrease the efficiency of neurotransmitter release and synaptic function, impairing behavior and cognition and conferring risk to ADHD. It should be noted however that the above hypothesis must be viewed with some caution since the current study did not test the expression of *SNAP25* in post-mortem samples derived from individuals with ADHD.

Recent biochemical analyses have shown that SNAP-25 amino acids (AA) 7–83 and 141–204 are essential motifs that are spontaneously assembled into helical SNARE complexes with Syntaxin1 and synaptobrevin 2 motifs [33]. *SNAP25* mutations introduced to the C terminal of the protein at AA positions 78, 81 and 202 resulted in a near elimination of exocytosis [34]. Furthermore, two alternative transcripts have been observed encoding two SNAP-25 protein isoforms. Variant 1 has 8 exons whereas variant 2 contains an alternative exon 5 as compared to variant 1, with the resulting isoform being of the same length but differing by 9 amino acids. Although we do not know which SNAP-25 isoforms are associated with ADHD, it is important to note that the SNAP-25 motif (AA 7–83 and 141–204) that is critical to SNARE formation and the exon 5 splice variant described above, all lie within the ADHD-associated region described herein. These observations further emphasize the importance of this region for the integrity of SNAP-25 and SNARE function and consequently for the development of ADHD.

In summary, this study provides support for the involvement of *SNAP25* as a susceptibility locus for ADHD. We hypothesize that the region between intron 3 and the 3UTR of *SNAP25* may harbor functional variants that confer risk to ADHD. Finally, we stress the importance of independent replication of our findings preferably in different ethnic samples.
